# Curly Kale (*Brassica oleracea var. Sabellica* L.) Biofortified with 5,7-Diiodo-8-quinolinol: The Influence of Heat Treatment on Iodine Level, Macronutrient Composition and Antioxidant Content

**DOI:** 10.3390/nu15224730

**Published:** 2023-11-09

**Authors:** Justyna Waśniowska, Teresa Leszczyńska, Aneta Kopeć, Ewa Piątkowska, Sylwester Smoleń, Joanna Krzemińska, Iwona Kowalska, Jacek Słupski, Ewelina Piasna-Słupecka, Katarzyna Krawczyk, Aneta Koronowicz

**Affiliations:** 1 Department of Human Nutrition and Dietetics, Faculty of Food Technology, University of Agriculture in Krakow, al. Mickiewicza 21, 31-120 Krakow, Poland; justyna.wasniowska@student.urk.edu.pl (J.W.); teresa.leszczynska@urk.edu.pl (T.L.); aneta.kopec@urk.edu.pl (A.K.); ewa.piatkowska@urk.edu.pl (E.P.); joanna.krzeminska@urk.edu.pl (J.K.); ewelina.piasna@urk.edu.pl (E.P.-S.); katarzyna.krawczyk@student.urk.edu.pl (K.K.); 2 Department of Plant Biology and Biotechnology, Faculty of Biotechnology and Horticulture, University of Agriculture in Krakow, al. Mickiewicza 21, 31-120 Krakow, Poland; sylwester.smolen@urk.edu.pl (S.S.); iwona.kowalska@urk.edu.pl (I.K.); 3 Department of Plant Product Technology and Nutrition Hygiene, Faculty of Food Technology, University of Agriculture in Krakow, al. Mickiewicza 21, 31-120 Krakow, Poland; jacek.slupski@urk.edu.pl

**Keywords:** curly kale, biofortification, thermal treatment, iodine, 5,7-diiodo-8-quinolinol, iodine bioavailability

## Abstract

Many disorders are a result of an inadequate supply of macronutrients and micronutrients in the diet. One such element is iodine. This study used curly kale (*Brassica oleracea var. Sabellica* L.) biofortified with the 5,7-diiodo-8-quinolinol iodine compound. The effect of the heat treatment on the chemical composition of the curly kale was studied. In addition, iodine bioavailability was evaluated in in vivo studies. Our investigation showed that iodine loss depends on the type of heat treatment as well as on the variety of kale. Curly kale biofortified with iodoquinoline had significantly higher iodine levels after thermal processing (steaming, blanching, boiling) than the vegetable biofortified with KIO_3_. Generally, steaming was the best thermal processing method, as it contributed to the lowest iodine loss in curly kale. The red variety of kale, ‘Redbor F_1_’, showed a better iodine stability during the heat treatment than the green variety, ‘Oldenbor F_1_’. The thermal treatment also significantly affected the dry matter content and the basic chemical composition of the tested varieties of the 5,7-diI-8-Q biofortified kale. The steaming process caused a significant increase in total carbohydrates, fiber, protein and crude fat content (‘Oldenbor F_1_’, ‘Redbor F_1_’), and antioxidant activity (‘Oldenbor F_1_’). On the other hand, boiling caused a significant decrease, while steaming caused a significant increase, in protein and dry matter content (‘Oldenbor F_1_’, ‘Redbor F_1_’). The blanching process caused the smallest significant decrease in ash compared to the other thermal processes used (‘Oldenbor F_1_’). A feeding experiment using Wistar rats showed that iodine from the 5,7-diI-8-Q biofortified kale has a higher bioavailability than that from the *AIN-93G* diet. A number of promising results have been obtained, which could form the basis for further research.

## 1. Introduction

Modern society is characterized by a growing awareness of the benefits of a healthy lifestyle and proper nutrition. Within this context, the vegetarian diet is becoming increasingly popular as one of the leading dietary choices, characterized by a reduction in the consumption of meat and, often, animal products. The increased interest in this dietary pattern is due to both ethical and health reasons. Previous research has shown that a vegetarian diet may be associated with a reduced risk of chronic diseases such as heart disease, high blood pressure, type 2 diabetes, and certain types of cancer [1,2].

The exclusion of meat and animal products in a vegetarian diet affects the nutritional balance by reducing the intake of saturated fats, cholesterol, and harmful substances found in certain meat products. At the same time, this diet can promote an increased intake of fiber, vitamins, minerals, and antioxidants from plant sources, which may contribute to beneficial health effects. However, there is one nutritional aspect that may require special attention in the context of a vegetarian diet—iodine. It occurs naturally as iodine salts in seaweeds, shellfish, and fish. Eliminating these products from a vegetarian diet may increase the risk of iodine deficiency. A major source of dietary iodine is iodized salt. The recommended dietary allowance (RDA) of iodine for an average adult is 150 µg/day [3]. The WHO recommends reducing salt intake to 5 g NaCl/person/day (provides 76.5–153.5 µg of iodine); thus, prevention through using iodized table salt is insufficient. Salt intake above the recommended amount increases the risk of cardiovascular disease and malignant tumors [4].

Iodine is a micronutrient essential for the proper functioning of the human body. It is responsible for the synthesis of the thyroid hormones thyroxine (T4) and triiodo-thyronine (T3), and its deficiency in the diet may lead to serious health consequences. Iodine performs a key role in the regulation of metabolic processes and in cardiovascular function, and it is also an essential element for the appropriate development of the body during prenatal life, childhood, and adolescence. Iodine deficiency (ID) is considered one of the causes of birth defects, and increased perinatal mortality including hypothyroidism, endemic goiter, learning disabilities, and cretinism [5]. The content of iodine in the human body is determined by the iodine content in the soil and, consequently, in plants and animal products [4]. Mountainous areas typically have iodine-deficient soil due to slow iodine cycling; thus, people who live there are more exposed to the risk of ID disorders [6]. Therefore, new alternative approaches to increase the iodine contribution to the human diet are being sought.

Several studies have investigated the possibility of using crops fertilized with iodine compounds [7,8,9,10]. This element is present in iodoquinolines, which are quinoline derivatives [11]. Vegetables enriched with iodine could be a good source of this micronutrient due to their relatively large consumption in the average diet; they may improve the body’s iodine saturation without the risk of overconsumption.

Curly kale (*Brassica oleracea var. Sabellica* L.) is one of the oldest cabbage-like plants. It contains significant amounts of minerals, carotenoids, vitamins (B-group, C, K), and dietary fiber. This vegetable is also a source of glucosinolates, of which some hydrolysis products have been proven to have anticancer properties. On the other hand, pro-goitrin, belonging to this group, after being transformed into goitrin in the human body, can bind iodine, preventing the synthesis of thyroid hormones. In the Centers for Disease Control’s study of 47 “powerful” fruits and vegetables, kale was ranked 15th (a serving containing ≥10% of 17 essential nutrients), meaning it is closely linked to reducing the risk of heart diseases and many other non-communicable diseases [8,12]. Kale is one of the vegetables that, as a result of biofortification, has the potential to accumulate high amounts of iodine [13].

It was shown, in our own pilot studies, that biofortification with 5,7-diiodo-8-quinolinol (5,7-diI-8-Q), among several other tested inorganic and organic iodine compounds, has satisfactory effectiveness for iodine enrichment of curly kale.

It was hypothesized that curly kale biofortified with 5,7-diI-8-Q is more resistant to iodine loss (in percentage) during the thermal processing of this vegetable compared to the vegetable biofortified with KIO_3_ and without biofortification; plus, iodine from the biofortified vegetable has a good bioavailability and absorption from the gastrointestinal tract.

The aim of this study was to determine the influence of the heat treatment on the nutritional value, i.e., iodine level, basic chemical composition, and selected antioxidants, in two cultivar varieties (cv.) of curly kale (*Brassica oleracea var. Sabellica* L.), i.e., ‘Oldenbor F_1_’ and ‘Redbor F_1_’, previously biofortified with 5,7-diI-8-Q and KIO_3_ (as a reference). In addition, the bioavailability of iodine from the biofortified kale was estimated in a feeding experiment with Wistar rats.

To the best of our knowledge, this study is the first to evaluate the effect of the heat treatment on the nutritional value, i.e., iodine content and chemical composition, of kale (*Brassica oleracea var. Sabellica* L.) cv. ‘Oldenbor F_1_’ and ‘Redbor F_1_’ biofortified with 5,7-diI-8-Q and the bioavailability of iodine in a feeding experiment with Wistar rats.

## 2. Materials and Methods

### 2.1. Synthesis of 5,7-Diiodo-8-quinolinol

The classical synthesis of 5,7-diiodo-8-quinolinol [14] was modified using microwave conditions that optimize the reaction solvent, time, and temperature. The synthesis of 5,7-diiodo-8-quinolinol was carried out as described by Krawczyk et al. [15].

### 2.2. Curly Kale Cultivation and Biofortification with KIO_3_ and 5,7-Diiodo-8-quinolinol

Hydroponic cultivation of curly kale (*Brassica oleracea var. Sabellica* L.) cv. ‘Olden-bor F_1_’ with green leaves and ‘Redbor F_1_’ with red leaves was carried out in spring 2022 in the Nutrient Film Technology system. The experiments were located in the Faculty of Biotechnology and Horticulture at the University of Agriculture in Kraków (Poland). The ‘dry hydroponic’ (NFT) method of growing plants without a substrate was used, with plants placed in holes in trays at 30 cm intervals. The plants were watered during the day and at night between 5.00 and 19.00 and between 1.00 and 2.00 h, respectively, for 1 min at 5 min intervals. The nutrient solution used to grow the curly kale contained micro- and macronutrients at concentrations (mg·L^−1^) of N 130, P 50, K 220, Mg 45, Ca 120, Fe 2, Mn 0.55, Zn 0.33, B 0.33, Cu 0.15, and Mo 0.05, and it was pH 5.70, adjusted with 38% nitric acid. The final EC of 2.0 mS-cm^−1^ was obtained. The following treatments were included in the experiment: control and application of KIO_3_ and 5,7-diI-8-Q at 10 µM concentration (each of the iodine compounds), which was started at the stage of 4–5 true leaves and was continued until the harvest of the plants. There were four replicates, 6 plants per replicate, and 24 plants per combination of each variety, yielding a total of 78 plants of each variety in the experiment. The plants were harvested at the stage of 13–14 true leaves after 63 and 70 days of cultivation.

### 2.3. Thermal Treatment of Curly Kale

On the day of the harvest, the leaves of the control group and of the iodoquinoline-enriched group were separated from the stems. This was performed to obtain the edible part. The leaves were then subjected to the heat treatments, i.e., steaming, blanching, and boiling.

The steaming process was carried out in a combi-steam oven (Retigo Orange Version 6 × GN1/1|O 611, Rožnov, Czech Republic). The curly kale leaves were placed on metal trays with one layer of this vegetable on each tray. They were then steamed at 100 °C for about 15 min. Steaming was continued until the kale was soft and easy to chew.

The blanching was carried out with distilled water using an electric cooker (Mastercook KE 2003 B Dynamic, Wroclaw, Poland). A portion of the curly kale was placed in an aluminum pot covered with layers of stainless steel. It was then flooded with water at 85 °C and left covered in the water for about 3–5 min. The curly kale was then strained and poured over with cold distilled water.

The boiling was carried out in distilled water using an electric cooker (Mastercook KE 2003 B Dynamic, Wroclaw, Poland). The curly kale was boiled in water in an aluminum pot covered with layers of stainless steel. The water was brought to boil and the curly kale was boiled for 20 min in 1 L of water, until ready to be consumed. The boiling process was carried out without a lid.

### 2.4. Chemical Composition

The contents of the components, such as dry matter, basic chemical compounds (dry matter, protein, crude fat, dietary fiber, total carbohydrates, digestible carbohydrates, ash), vitamin C, total carotenoids, total polyphenols, and free radical quenching capacity of ABTS *+, after the thermal treatment, were calculated using the true retention method (%TR). This method requires data on the weight of the food before and after cooking, as well as on the nutrient contents of the raw and cooked food [16].

TR [%] = (Nc · Gc)/(Nr · Gr) · 100

Nc = nutrient content per gram of heat-treated food;

Gc = gram of heat-treated food;

Nr = nutrient content per gram of raw food;

Gr = gram of food before heat treatment.

The content of vitamin C was measured, and extracts were prepared in raw material to determine the content of total polyphenols, total carotenoids, and the antioxidant activity.

The contents of total iodine, total protein, crude fat, total carbohydrates, total dietary fiber, and ash were analyzed in lyophilized material.

#### 2.4.1. Lyophilization of the Analytical Material

The raw and heat-treated curly kale leaves (steaming, blanching, boiling) were frozen at −20 °C and freeze-dried in a Christ Alpha 1–4 freeze dryer (Martin Christ Gefriertrocknungsanlagen GmbH, Osterode am Harz, Germany). The freeze-dried leaf samples were ground in a laboratory grinder (FRITSCH Pulverisette 14, Idar-Oberstein, Germany) and stored in sealed polyethylene bags in refrigerated conditions until they were analyzed.

#### 2.4.2. Determination of Dry Matter Content

The procedure by the Association of Official Analytical Chemists (AOAC) was used to determine the dry matter (g·kg^−1^) content of all curly kale samples (‘Oldenbor F_1_’, ‘Redbor F_1_’) at 105 °C.

#### 2.4.3. Determination of Total Iodine

To assess the iodine content of raw and heat-processed kale (steaming, blanching, boiling), samples (each sample contained 0.2 g lyophilized curly kale) were extracted with tetra-methylammonium hydroxide (TMAH). The preparation of samples for iodine determination was carried out as described by Smoleń [8]. The iodine content of the analyzed samples was then measured with a triple quadruple spectrometer (iCAP TQ ICP-MS ThermoFisher Scientific, Bremen, Germany) using the technique of inductively coupled plasma mass spectrometry (ICP-MS/MS). The results were expressed on a fresh weight basis (mg·kg^−1^).

#### 2.4.4. Basic Chemical Composition

The procedures of the Association of Official Analytical Chemists (AOAC) were used to determine the basic chemical composition. The results were expressed on a fresh weight basis (g·kg^−1^). The total protein content was assessed using the Kjeldahl method (AOAC procedure no. 950.36). The crude fat was estimated according to the Soxhlet extraction method (AOAC procedure no. 935.38). The total dietary fiber was assessed using a commercially available test kit (AOAC procedure no. 991.43). The ash content of the kale was determined by burning the samples in a muffle furnace (AOAC procedure no. 930.05). The content of digestible carbohydrates was determined using the following formula: Digestible carbohydrates = 100 − (total protein + crude fat + ash + total dietary fiber content).

#### 2.4.5. Determination of Vitamin C, Total Carotenoids, Total Polyphenol Content, and Free Radical Quenching Capacity of ABTS *^+^

The ascorbic acid (AA) and dehydroascorbic acid (DHA) contents were analyzed using a Beckman PA 800 Plus capillary electrophoresis (CE) system according to the method described by Smoleń [7].

The material for the analyses was prepared via homogenization (20 g samples in 80 cm^3^ of 2% oxalate acid; puriss. p.a., Avantor Performance Materials, Radnor, PA, USA) and centrifuging (15 min at 4500 rpm, 5 °C). The supernatants were analyzed using a PA 800 Plus capillary electrophoresis system (Beckman Coulter, Indianapolis, IN, USA) with diode array detector (DAD) detection, after filtration through a 0.25 μm cellulose acetate membrane filter. Capillaries of 50 μm inner diameter and 365 μm outer diameter were used, as well as those with a total length of 50 cm (40 cm to the detector). A negative power supply of 25 kV was applied. The running buffer solution was prepared. It contained 15 mM Na2B4O7 (puriss. p.a., Sigma-Aldrich, St. Louis, MO, USA), 0.2 mM cetyltrimethylammonium bromide (CTAB) (puriss. p.a., Sigma-Aldrich) (pH 8.80), and 30 mM NaH2PO4 (puriss. p.a., Avantor Performance Materials). The results were expressed on a fresh weight basis (g·kg^−1^).

The total carotenoid content was determined with a colorimetric method according to PN-90/A-75101/12 [17]. In line with this method, the sample was extracted with a hexane–acetone mixture and the absorbance was read at a wavelength of λ = 450 nm. The results were expressed on a fresh weight basis (g·kg^−1^).

Total polyphenolic content and antioxidant activity were tested in ethanol extracts. For the preparation of ethanol extracts, −0.7 g of lyophilized curly kale was placed in a conical flask and 100 mL of 70% ethanol was added. The flasks were shaken in a water bath shaker for 2 h at 40 °C. The resulting extracts were filtered.

The total polyphenolic content was determined via spectroscopy using the Folin–Ciocalteu (F-C) reagent, with gallic acid (GA) as the standard, to determine the curve. The method used is based on the reaction of polyphenolic compounds present in the test sample with the components of the Folin–Ciocalteu reagent, i.e., sodium molybdate and sodium tungstate. The results were expressed on a fresh weight basis (g·kg^−1^).

The free radical quenching capacity of ABTS *^+^ was determined using the ABTS method [17]. Trolox solution (standard range of 0.0–10 μmol) was used and expressed as micromoles of Trolox equivalent per gram of fresh weight (TEAC).

### 2.5. Assessment of Iodine Fortified Kale for Consumer Health and Safety

The iodine content in 50 and 100 g of curly kale leaves and the percentage of the recommended dietary allowance of iodine (% RDA-I) were calculated (Table 2). The RDA-I was based on the World Health Organization (WHO) recommendations for children over 12 years of age and adults, i.e., 150 µg/person/day [18]. The results were expressed on a fresh weight basis (g·kg^−1^).

The consumer safety of iodine-enriched curly kale leaves was assessed using hazard quotient (HQ) values, which describe the risk to human health from the iodine intake from the consumption of heat-treated kale leaves. The HQ-I values were calculated according to the following equation: HQ = ADD/RfD, where ADD is the average daily intake of iodine (mg of I per kg body weight per day) and RfD is the recommended upper dietary intake limit of iodine [19]. The average daily dose (ADD) was calculated as ADD = (MI-CF-DI)/BW, where MI is the iodine concentration in the thermally treated curly kale leaves (mg·kg^−1^ f.w.), CF is the conversion factor from fresh to dry weight of the plant samples (dry-to-fresh weight ratio; average 0.162), DI is the daily iodine intake (0.1 kg), and BW is the assumed body weight (70 kg). The assumed RfD value for iodine was 1100.

### 2.6. Animal Study

Five-week-old male Wistar rats (n = 24) with an average body weight of 129 ± 10 g were obtained from Animal Husbandry at the Animal Jagiellonian University Medical College, Kracow, Poland.

The First Local Ethical Committee approved the experimental procedures for Animal Experiments in Krakow (Poland, res. no. 568/2021).

The rodents were acclimated on a standard laboratory chow for one week prior to the experiment. At the end of the acclimatization period, the rodents were randomly divided into five experimental groups (n = 8). Experimental diets were prepared based on the *AIN-93G* diets [20]. The detailed compositions of the diets can be found in Table 1 below.

Group 1 was fed the *AIN-93G* (C) diet, the mineral mix containing iodine in line with Reeves’ recommendation [21]. For group 2 (CO) with the control curly kale ‘Oldenbor F1’ and for group 4 (CR) with the control curly kale ‘Redbor F_1_’, both diets were prepared with a mineral mixture with iodine. In the diet containing biofortified curly kale (group 3, BO diet with biofortified 5,7-diI-8-Q raw curly kale ‘Oldenbor F_1_’; and group 5, BR diet biofortified with 5,7-diI-8-Q raw curly kale ‘Redbor F_1_’), the only source of iodine was kale (mineral mixture did not contain iodine) (Table 1).

The rodents were housed separately in metabolic cages made of stainless steel in the first and eighth week of the experiment, at 21 °C and a 12/12 h light/dark cycle. The animals had free access to deionized distilled water throughout the experiment. Consumption of the experimental diets was recorded daily. Rats were housed in conventional open-top cages with two or three animals per cage for the remaining weeks. Body weight gain was recorded weekly throughout the experiment. The duration of the experiment was 8 weeks. The results were expressed on a fresh weight basis (µg·L^−1^) (urine) and dry weight basis (g·kg^−1^) (feces).

#### Iodine Content in Urine and Feces

Urine and feces were collected during the first and the eighth weeks of the experiment to assess iodine excretion. Collected urine samples were made equal before analysis. All samples were frozen and stored at −20 °C. Urine samples were thawed and mixed. Then, 4.8 mL of urine was collected into a PP tube, and 0.2 mL of 25% TMAHu was added and incubated with this reagent. Subsequently, the samples were mixed and diluted 200× in water redistilled three times. Then, an analysis was performed on ICP-MS/MS according to the same method as described in Section 2.4.3.

### 2.7. Statistical Analysis

Statistical analysis was performed using Statistica 13.1 PL. All experiments were performed with at least four replications. A three-way analysis of variance was used with the main effects being as follows: variety (‘Oldenbor F_1_’, ‘Redbor F_1_’), enrichment (5,7-diiodo-8-quinolinol), heat treatment (steaming, blanching, boiling), with all possible interactions. It was also used to analyze the iodine content, dry matter, basic chemical composition, vitamin C, total carotenoids, total polyphenols, and free radical quenching capacity of ABTS *+ of kale. A two-way analysis of variance was used with the main effects being as follows: variety (‘Oldenbor F_1_’, ‘Redbor F_1_’), enrichment (5,7-diiodo-8-quinolinol) used in the animal experiment. Statistically significant differences were assessed using a post hoc Tukey’s test; *p* values ≤ 0.05 were considered statistically significant.

## 3. Results

### 3.1. Iodine Content

**Figure 1:**
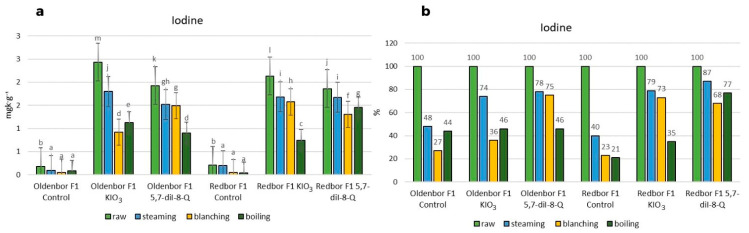
Iodine contents in raw and heat-treated biofortified curly kale (steaming, blanching, boiling). (**a**) Contents of iodine in two curly kale cultivars (‘Oldenbor F_1_’ and ‘Redbor F_1_’) depending on the thermal treatment. Means followed by the same letters for treatments for each curly kale variety cultivar are not significantly different (*p* > 0.05). (**b**) Percentage difference in iodine content of biofortified curly kale leaves given temperature treatment compared to raw biofortified curly kale.

The use of 5,7-diiodo-8-quinolinol (5,7-diI-8-Q) in the biofortification process effectively increased the iodine level in the plant (Figure 1). Culinary thermal treatments showed the following effects on the iodine content of the biofortified curly kale (‘Oldenbor F_1_’, ‘Redbor F_1_’). The lowest (although still significant) decrease in iodine content was observed in the ‘Oldenbor F_1_’ variety in kale biofortified with KIO_3_ subjected to the steaming process, while in ‘Redbor F_1_’ (biofortified with KIO_3_, 5,7-diI-8-Q), the decrease was significantly higher but still resulted in the lowest iodine content loss in kale compared to other thermal processes. The steaming process resulted in the lowest statistically significant (*p* ≤ 0.05) loss of iodine in the two biofortified (KIO_3_, 5,7-diI-8-Q) kale varieties (Figure 1a).

The blanching of biofortified (KIO_3_) curly kale resulted in the lowest iodine levels in curly kale of the ‘Oldenbor F_1_’ variety (Figure 1a) compared to 5,7-diI-8-Q-enriched curly kale in both varieties and in the ‘Oldenbor F_1_’ variety (biofortified with 5,7-diI-8-Q) (Figure 1a).

In the boiling process, the largest decrease was observed in ‘Redbor F_1_’ (biofortified with KIO_3_) compared to the ‘Redbor F_1_’ variety (biofortified with 5,7-diI-8-Q) and the ‘Oldenbor F_1_’ variety (biofortified with KIO_3_, 5,7-diI-8-Q) (Figure 1a). Among the thermal processes used, the smallest (statistically significant *p* ≤ 0.05) decrease in iodine content was observed after steaming, while the largest (statistically significant *p* ≤ 0.05) decrease was observed after boiling the vegetables.

The lowest percentage decrease in iodine content after the steaming process was recorded in the curly kale variety biofortified with 5,7-diI-8-Q (‘Redbor F_1_’); the iodine content of heat-treated curly kale was 87% of that in raw biofortified curly kale (Figure 1b). However, the largest percentage decrease was also observed in the same variety biofortified with KIO_3_, but after boiling; the iodine content of heat-treated curly kale was 35% of that in raw curly kale (Figure 1b). Among the thermal processes used, the steaming process caused the smallest decrease in iodine content in both biofortified (KIO_3_, 5,7-diI-8-Q) varieties. In contrast, the largest percentage decrease in the ‘Oldenbor F_1_’ variety was caused by the blanching process (up to 36%) in the product biofortified with KIO_3_ and by the boiling process (up to 46%) in the product biofortified with 5,7-diI-8-Q (Figure 1b). In the ‘Redbor F_1_’ variety, the largest percentage decrease was caused by the boiling process (up to 35%) (biofortified with KIO_3_) and the blanching process (up to 68%) (biofortified with 5,7-diI-8-Q) (Figure 1b).

### 3.2. Dry Matter Content and Basic Chemical Composition

**Figure 2:**
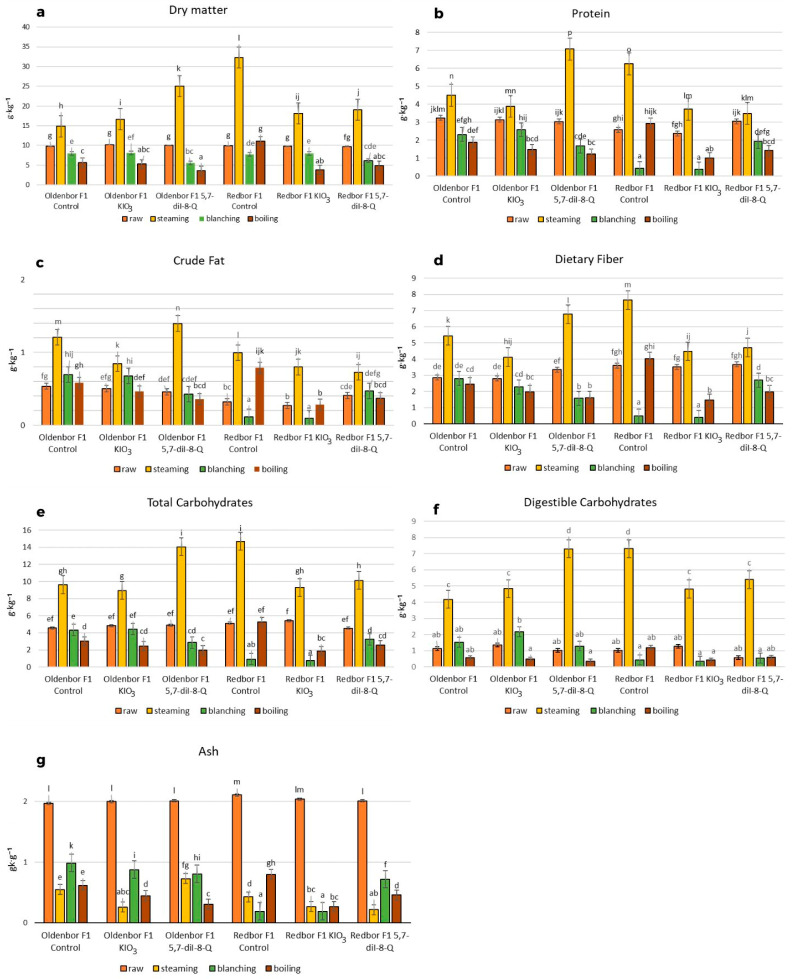
Dry matter contents and basic chemical compositions of biofortified curly kale before and after heat treatment (f.w). Contents of dry matter (**a**), protein (**b**), crude fat (**c**), dietary fiber (**d**), total carbohydrates (**e**), digestible carbohydrates (**f**), and ash (**g**) in two curly kale cultivars (‘Oldenbor F_1_’, ‘Redbor F_1_’) under thermal treatment. Means followed by the same letters for treatments separately for each curly kale variety cultivar are not significantly different (*p* > 0.05).

Heat treatment of curly kale biofortified with 5,7-diiodo-8-quinolinol had a significant effect on the dry matter content and the chemical composition compared to raw curly kale (Figure 2).

The highest level of dry matter content was determined in the biofortified (5,7-diI-8-Q) ‘Oldenbor F_1_’ variety after the steaming process when compared to raw curly kale (Figure 2a). The dry matter content in ‘Oldenbor F_1_’ curly kale enriched with iodoquinoline was significantly (*p* ≤ 0.05) higher compared to that biofortified with KIO_3_. In addition, the dry matter content of the biofortified (5,7-diI-8-Q) ‘Oldenbor F_1_’ variety was statistically (*p* ≤ 0.05) higher than that of the biofortified (5,7-diI-8-Q) ‘Redbor F_1_’ variety after steaming.

The steaming process significantly (*p* ≤ 0.05) increased the protein content of control kale (biofortified with KIO_3_) of both varieties and kale (‘Oldenbor F_1_’) biofortified with 5,7-diI-8-Q (Figure 2b). In contrast, boiling caused the highest significant (*p* ≤ 0.05) decrease in the protein content of biofortified (KIO_3_, 5,7-diI-8-Q) ‘Oldenbor F_1_’ and ‘Redbor F_1_’ (5,7-diI-8-Q) curly kale varieties.

The highest crude fat content was found in the biofortified (5,7-diI-8-Q) ‘Oldenbor F_1_’ curly kale after steaming. The level of crude fat was higher in the biofortified (5,7-diI-8-Q) ‘Oldenbor F_1_’ variety compared to the enriched (5,7-diI-8-Q) ‘Redbor F_1_’ variety during the steaming process. The lowest level (*p* ≤ 0.05) of crude fat was obtained in curly kale after boiling in the ‘Oldenbor F_1_’ variety and after blanching in the ‘Redbor F_1_’ variety.

There was a significant (*p* ≤ 0.05) increase in total dietary fiber in both varieties enriched with KIO_3_ and 5,7-diI-8-Q after the steaming process compared to the raw vegetable, but the levels differed significantly (*p* ≤ 0.05) only between varieties in kale biofortified with 5,7-diI-8-Q (Figure 2d). There was a significant decrease in fiber content in ‘Redbor F_1_’ (biofortified KIO_3_ and 5,7-diI-8-Q) and ‘Oldenbor F_1_’ (biofortified 5,7-diI-8-Q) after blanching and boiling of kale.

The total carbohydrate content increased significantly (*p* ≤ 0.05) and was the highest after the steaming process in both enriched varieties as compared to biofortified blanched and boiled curly kale (Figure 2e). The level of the total carbohydrates was significantly higher in the ‘Oldenbor F_1_’ variety (enriched with 5,7-diI-8-Q) compared to ‘Redbor F_1_’. The lowest levels of total carbohydrates were found in ‘Oldenbor F_1_’ (both biofortified with KIO_3_ and 5,7-diI-8-Q) and ‘Redbor F_1_’ (biofortified with 5,7-diI-8-Q) after the boiling process in comparison with raw kale (*p* ≤ 0.05). The differences in the total carbohydrate content between the 5,7-diI-8-Q-enriched and KIO_3_-treated varieties were not significant (*p* > 0.05) other than for the ‘Redbor F_1_’ variety.

The digestible carbohydrate content increased significantly after the steaming process in both biofortified varieties (KIO_3_ and 5,7-diI-8-Q) as compared to biofortified raw curly kale (Figure 2f). The level of the digestible carbohydrates was significantly (*p* ≤ 0.05) different between the enhancements in the biofortified variety ‘Oldenbor F_1_’ (KIO_3_ and 5,7-diI-8-Q). The lowest level of digestible carbohydrates was recorded after boiling in the two biofortified (KIO_3_ and 5,7-diI-8-Q) varieties and after blanching in the biofortified (KIO3 and 5,7-diI-8-Q) ‘Redbor F_1_’ variety and in the biofortified (5,7-diI-8-Q) ‘Oldenbor F_1_’ variety.

The highest decreased (*p* ≤ 0.05) ash content was observed after the steaming process in biofortified ‘Oldenbor F_1_’ (KIO_3_) and ‘Redbor F_1_’ (5,7-diI-8-Q) varieties and after boiling (‘Oldenbor F_1_’; biofortified with 5,7-diI-8-Q) and blanching processes (‘Redbor F_1_’; biofortified with KIO_3_) compared to biofortified raw curly kale. The ash level after the steaming process of the (5,7-diI-8-Q) ‘Oldenbor F_1_’ variety was significantly higher compared to the (5,7-diI-8-Q) ‘Redbor F_1_’ variety. The highest ash content was observed after blanching in the biofortified ‘Oldenbor F_1_’ (KIO_3_, 5,7-diI-8-Q) variety and the ‘Redbor F_1_’ variety biofortified with 5,7-diI-8-Q compared to raw curly kale. The ash levels after the above thermal process differed significantly between varieties and enrichments (Figure 2g).

### 3.3. Vitamin C, Total Carotenoids, Total Polyphenol Content, and Free Radical Quenching Capacity of ABTS *^+^

**Figure 3:**
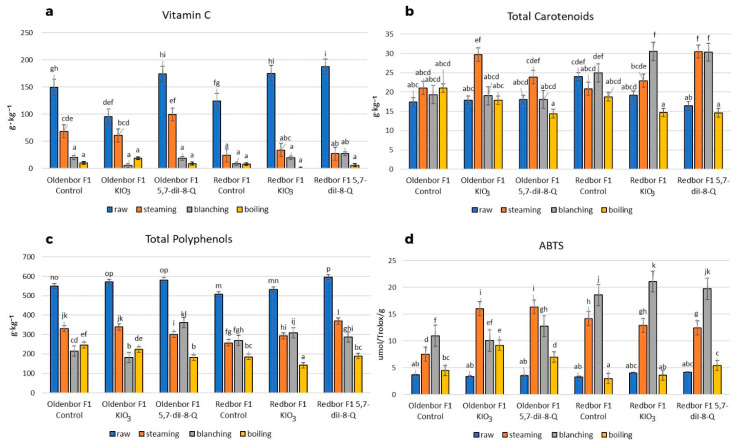
Vitamin C, total carotenoids, total polyphenol content, and free radical quenching capacity of ABTS *^+^ (f.w). Contents of vitamin C (**a**), total carotenoids (**b**), total polyphenols (**c**), and ABTS *^+^ (**d**) in two curly kale cultivars (‘Oldenbor F_1_’, ‘Redbor F_1_’) under thermal treatment. Means followed by the same letters for treatments for each curly kale variety cultivar are not significantly different (*p* > 0.05).

Heat treatment (boiling, steaming, blanching) of both curly kale varieties biofortified with an iodine compound (5,7-diI-8-Q) had a significant (*p* ≤ 0.05) effect on vitamin C, total polyphenol content, and antioxidant activity (Figure 3).

The steaming process caused the smallest decrease in the content of vitamin C in the biofortified (KIO_3_, 5,7-diI-8-Q) ‘Oldenbor F_1_’ variety compared to the raw curly kale (Figure 3a). The vitamin C content in ‘Oldenbor F_1_’ curly kale biofortified with 5,7-diI-8-Q was statistically (*p* ≤ 0.05) higher than that in KIO_3_-enriched curly kale. The greatest decrease in vitamin C content was observed in the biofortified (KIO_3_, 5,7-diI-8-Q) ‘Redbor F_1_’ variety treated with boiling, blanching, and steaming, as well as in the biofortified (KIO_3_, 5,7-diI-8-Q) ‘Oldenbor F_1_’ variety treated with boiling and blanching compared to raw curly kale. 

There was a significant (*p* ≤ 0.05) effect (most often) of the curly kale variety and heat treatment on the carotenoid content (Figure 3b). In all samples, steaming process increased (*p* ≤ 0.05) the carotenoid content in the ‘Redbor F_1_’ variety biofortified with 5,7-diI-8-Q as compared to that biofortified with KIO_3_. Boiling had no significant effect on the carotenoid content in either of the varieties enriched with KIO_3_ and 5,7-diI-8-Q. Differences in total carotenoids were not significant (*p* > 0.05) within cultivar or enrichment.

The steaming process caused the smallest decrease in total polyphenols in ‘Redbor F_1_’ (5,7-diI-8-Q) and ‘Oldenbor F_1_’ (KIO_3_), while in ‘Oldenbor F_1_’ (5,7-diI-8-Q), blanching had the same effect. The differences between the curly kale varieties were significant (*p* ≤ 0.05) after the steaming and blanching processes. Boiling caused the greatest decrease in total polyphenols in both biofortified (5,7-diI-8-Q) varieties (Figure 3c).

The steaming process caused the highest increase in antioxidant activity in the biofortified ‘Oldebor F_1_’ variety compared to raw curly kale. Differences between curly kale enriched with KIO_3_ and 5,7-diI-8-Q were not statistically significant (*p* > 0.05) (Figure 3d). In contrast, in the biofortified curly kale ‘Redbor F_1_’, the blanching process caused the highest increase in antioxidant activity, but the differences between curly kale enriched with KIO_3_ and 5,7-diI-8-Q were not significant (*p* > 0.05). The boiling process caused the lowest antioxidant activity in both varieties of biofortified (KIO_3_, 5,7-diI-8-Q) curly kale.

### 3.4. Assessment of Iodine in Biofortified Curly Kale for Consumer Health and Safety

Thermal processes acting on biofortified curly kale (steaming, blanching, boiling) resulted in a statistically significant (*p* ≤ 0.05) increase in the coverage of the recommended dietary allowance (RDA-I) for adult iodine intake in 50 and 100 g portions of this kind of vegetable (Table 2). For both varieties of the curly kale, steaming resulted in the lowest loss of iodine. The hazard quotient (HQ) value is intended to inform consumers about the food safety. If the HQ value is higher than 1, adverse health effects are possible. Heat treatment (steaming, blanching, boiling) caused a statistically significant (*p* ≤ 0.05) increase in the HQ, but its value did not exceed 1 for either curly kale variety (‘Oldenbor F_1_’, ‘Redbor F_1_’). The recommended daily intake of iodine per 50 g and 100 g portion of biofortified and heat-treated curly kale was higher in both varieties compared to the control group. The highest amount of this element was provided by biofortified and steamed curly kales.

Table 2 shows results only for kale (‘Oldenbor F_1_’, ‘Redbor F_1_’) biofortified with 5,7-diI-8-Q and without biofortification, when subjected to thermal treatment (steaming, blanching, boiling), because after heat treatment, iodine was better retained in this kind of vegetable than in vegetables biofortified with KIO_3_.

### 3.5. Bioavailability of Iodine in Animal Study

The research on iodine bioavailability was carried out only for kale (‘Oldenbor F_1_’, ‘Redbor F_1_’) biofortified with 5,7-diI-8-Q because of the limited number of animals in the study (according to the 3R rule).

**Figure 4:**
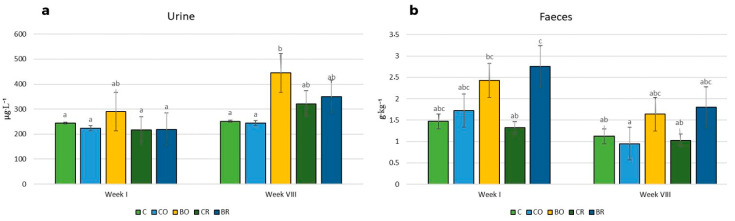
Concentrations of iodine in urine and feces of Wistar rats. Contents of urine (**a**), contents of feces (**b**); *C* control diet (*AIN-93G*), CO diet containing control curly kale ‘Oldenbor F_1_’, BO diet containing biofortified curly kale ‘Oldenbor F_1_’, CR diet containing control curly kale ‘Redbor F_1_’, BR diet containing biofortified curly kale ‘Redbor F_1_’. Means followed by the same letters for treatments for each kale variety cultivar are not significantly different (*p* > 0.05).

The highest urinary iodine excretion in the first and the eighth weeks was determined in the group of rats fed the BO diet compared to the other experimental groups (Figure 4). In addition, the lowest iodine levels were measured in the urine of rats fed the iodine-free diet (C), the non-biofortified curly kale ‘Oldenbor F_1_’ (CO) in both weeks, and the non-biofortified curly kale (‘Redbor F_1_’) diet (CR) and the biofortified diet (BR) in the first week. The highest iodine excretion in the first week was found in the feces of rats fed the BR diet and the lowest in rats fed the CR diet. In the eighth week, the lowest iodine excretion was found in the feces of rats fed the CO, CR, and C diets compared to the iodine concentration in the feces of rodents fed the BO and BR diets (Figure 4).

## 4. Discussion

**Figure 5:**
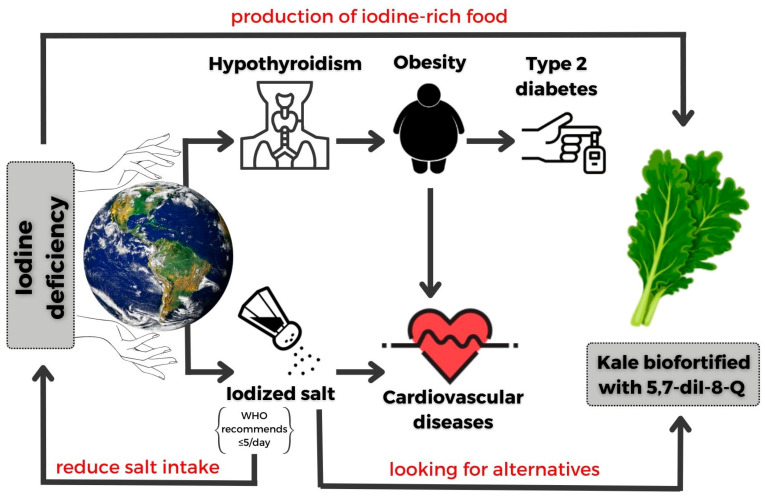
Consequences of iodine deficiency and an alternative to iodine-deficiency prevention (original artwork).

The World Health Organization has identified the biofortification of food as a suitable strategy to prevent micronutrient and macronutrient deficiencies. Currently, the iodine biofortification includes commonly consumed products such as cereals, vegetable oils, water, salt, and sugar. Among crops, lettuce, carrots, spinach, and cabbage are also good targets for iodine biofortification. In general, leafy vegetables are more convenient for iodine intake because they are consumed both raw and after heat treatment, and they are easy to fortify via foliar application. Iodine deficiency can be a significant challenge for those following a vegetarian diet. Therefore, this kind of diet leaves people heavily reliant on iodine from alternative sources, such as enriched vegetables (Figure 5).

Cruciferous vegetables, including curly kale, are a source of glucosinolates, non-nutritional ingredients that, on the one hand, show broad health-promoting properties, and on the other, hinder the absorption of iodine. Myrosinase, an enzyme that breaks down glucosinolates into their active anticancer forms, is activated when the vegetables are chopped [22,23]. From this point of view, an additional intake of iodine with biofortified cabbage-like vegetables, especially when heat-treated, would be beneficial for the human body because it could improve the amount of iodine bioavailability.

In the available literature, there are few data on the heat treatment of iodine-enriched vegetables and, therefore, a small list of literature sources is referred to in the discussion.

In our results, the content of the listed components was recalculated in relation to the mass balance, as presented in the Materials and Methods chapter. After the blanching and boiling processes, with the water absorption, the fresh weight increased from 8 to 35% or from 13 to 59%, respectively, but after the steaming process, it decreased from 3 to 12%. Therefore, the results were based on the final fresh weight of the product. Meanwhile, the authors cited herein reported changes in the composition due to the heat treatment per unit of dry or fresh matter, and few considered mass balance. Therefore, it is difficult to compare our results with those of the other authors.

The curly kale variety also influences the quantity of iodoquinoline-enriched curly kale. Thanks to the iodoquinoline biofortification, the curly kale under thermal treatment had a lower iodine loss (in percentage) than the curly kale without biofortification with 5,7-diI-8-Q. It follows that iodoquinoline has a protective function for iodine during thermal treatment. A significantly higher content of iodine in biofortified curly meant that even after the heat treatment, these vegetables contained a significantly higher amount of iodine than fresh ones non-biofortified with 5,7-diI-8-Q or even KIO_3_. The lowest iodine loss rates of 22 and 13% occurred after steaming for each variety, respectively, for ‘Oldenbor F_1_’ and ‘Redbor F_1_’ (biofortified with 5,7-diI-8-Q) compared to the raw curly kale. The boiling process resulted in iodine loss rates of 54% (‘Oldenbor F_1_’ variety biofortified with 5,7-diI-8-Q) and 33% (‘Oldenbor F_1_’ variety biofortified with 5,7-diI-8-Q) compared to raw curly kale. The study by Li et al. [9] on celery biofortified with KIO_3_ showed much lower iodine loss rates of only 1% and less than 15% after boiling for 2 and 30 min, respectively. In the Comandini et al. [24] study, cooking caused the greatest iodine loss depending on the vegetable variety, i.e., around 7.56–36% for potatoes, tomatoes, and carrots. Kapusta-Duch et al. [25] reported that the iodine content of KI biofortified carrot root decreases under thermal treatment (boiling in water). Kiferle et al. [26] showed that the cooking process also caused iodine loss in peeled or unpeeled tomatoes enriched with KI and KIO_3_. Iodine loss is not only dependent on the cooking time but also on the form of the plant product after processing (e.g., pulp), as reported by Salau et al. [27]. The range of iodine loss in the above studies in the used products was between 30% and 50%. Comandini et al. [24], in a study on iodine-biofortified potatoes, tomatoes, and carrots (method for enriching crop with iodine during cultivation on soil, comprising spraying the vegetables with a solution of an iodine salt with a concentration of iodine from 1 to 50 g/l), also showed the highest iodine loss from vegetables compared to the cooking process. The extraction of part of the iodine into the water caused the highest iodine loss during boiling. In order to reduce the loss of iodine during cooking, it is recommended to sprinkle salt on the food after cooking instead of adding salt during the cooking process [28,29]. However, salting food is not beneficial as excess salt contributes to cardiovascular disease. The WHO recommends an intake of up to 5 g/day, a dose easily exceeded as salt is found in many daily consumed products, e.g., bread and meat products [30]. Curly kale biofortified with 5,7-diI-8-Q can bring additional benefits by providing the deficient element, iodine, especially for people on vegetarian diets that exclude animal products as sources of iodine (leaving, e.g., seaweeds, shellfish, and fish). In addition, an adequate amount of iodine-biofortified vegetables in the diet and reducing the intake of the salt will help to reduce the risk of cardiovascular disease and can ensure an appropriate iodine intake, which is needed for the proper functioning of the thyroid gland. The thyroid gland needs iodine to produce thyroid hormones, which regulate metabolism. Iodine deficiency can cause hypothyroidism, which slows the metabolism and leads to weight gain and obesity [31]. Some patients develop what is known as android obesity, which is characterized by the accumulation of excess fatty tissue in the abdominal area [1,32]. The above information demonstrates the importance of an appropriate diet and the associated supply of sufficient iodine to the body; thus, it is important to look for new solutions, such as vegetable biofortification.

An important topic for research is the effect of 5,7-diI-8-Q biofortification and thermal processes on the basal composition of curly kale, as this will allow changes in the composition to be investigated and it will determine which process is the most beneficial. This issue is crucial as kale is consumed raw as well as after heat treatment.

A significant decrease in the dry matter content after boiling and blanching processes in both curly kale varieties (‘Oldenbor F_1_’,’Redbor F_1_’) biofortified with 5,7-diI-8-Q could be caused by the transfer of soluble components to water, with the exception of fat, which is insoluble in water. A significant increase in dry matter content after the steaming process in both curly kale varieties (‘Oldenbor F_1_’ ‘Redbor F_1_’) biofortified with 5,7-diI-8-Q may be due to the fact that in the steaming process, water evaporates from the product and the components generally remain. This results in a higher content of protein, crude fat, dietary fiber, and total and digestible carbohydrates in the steamed plant mass unit compared to the raw plant.

The increased protein and crude fat contents of curly kale (‘Oldenbor F_1_’, ‘Redbor F_1_’) biofortified with 5,7-diI-8-Q are beneficial in a vegetarian diet. Plant protein performs a key role when the meat and fish are excluded. For many people, a vegetarian diet provides sufficient protein while helping to reduce the intake of saturated animal fats and may have health benefits such as reducing the risk of obesity or heart disease [33,34]. Other researchers also showed an increase in fat content after cooking (broccoli, cauliflower) [35,36]. Ayaz et al. [37] showed that consuming kale leaves in winter would meet part of the requirement for omega-3 fatty acids, which have a documented good effect on health.

Dietary fiber is an important non-nutrient component found in plants that has many beneficial effects on human health, widely described in the literature [38,39,40,41,42]. There are two main types of dietary fiber: soluble and insoluble. Both types have different properties and react differently to thermal treatments such as steaming, blanching, and boiling. The steaming process of curly kale (‘Oldenbor F_1_’, ‘Redbor F_1’_) biofortified with 5,7-diI-8-Q resulted in an increase in dietary fiber content, whereas blanching and boiling resulted in its decrease. The decrease in fiber content after blanching or boiling can be explained by the leaching of its soluble part into the water. There is, therefore, also a reduction in the level of total carbohydrates, as demonstrated in our studies on curly kale (‘Oldenbor F_1_’, ‘Redbor F_1_’) biofortified with 5,7-diI-8-Q. In a study by Svanberg and Nyman [43], a decrease (about 90%) in total carbohydrate in carrots was also observed. In contrast, Andersson et al. [44] showed that the total carbohydrate content in artichokes, parsnips, and beetroot increased after boiling.

Our study showed a decrease in ash content after all heat treatments (stemming, blanching, boiling) in both kale varieties (‘Oldenbor F_1_’, ‘Redbor F_1_’) biofortified with 5,7-diI-8-Q compared to raw kale. During the mentioned thermal processes, volatile salts contained in the vegetables evaporate and water-soluble salts pass into the decoction [45].

The antioxidant activity of vitamin C, total carotenoids, and total polyphenols is based on their ability to neutralize free radicals that can damage cells and tissues in the body. In the thermal treatments carried out (steaming, blanching), an increase in the oxidative activity in curly kale (‘Oldenbor F_1_’, ‘Redbor F_1_’) biofortified with 5,7-diI-8-Q was found. Kapusta-Duch et al. [46] found that cooking reduced the antioxidant activity by only 0.52%. In contrast, in research by Sikora and Bodziarczyk [47], the loss of antioxidant activity during boiling of curly kale was much higher (62%). The vegetables studied by the cited scientists were not biofortified. The increase in the antioxidant activity in our study (despite vitamin C and total polyphenol decreases) may be due to the increased levels of carotenoids and other active compounds not assessed in the study, probably caused by the content of iodoquinoline [48].

In this study, it was found that the steaming process resulted in the smallest decrease in the vitamin C content in curly kale (‘Oldenbor F_1_’, ‘Redbor F_1_’) biofortified with 5,7-diI-8-Q. The reported loss was lower than those in other authors’ publications [47,49], who noted a decrease in this vitamin from 78 to 89%. The different amounts of loss could depend on the degree of grinding of the product, the temperature used, the length of the exposure to this temperature, the proportion of the vegetable’s weight to the amount of waters and the method of hydrothermal processing (traditional cooking, microwave cooking, etc.) [10,28,50].

Kurilich et al. [51] claim that curly kale has the highest carotene content of all Brassica vegetables (600 mg/100 g). In kale varieties biofortified with 5,7-diI-8-Q, when subjected to heat treatment, i.e., steaming (‘Oldenbor F_1_’, ‘Redbor F_1_’) and blanching (‘Redbor F_1_’), the total carotenoid content increased, but in the boiling process, it decreased. On the other hand, Gębczyński [52] noted decreases in the carotenoid content after blanching and cooking of 6 and 4%, respectively, compared to raw carrots. Nartea et al. [53] showed that the carotenoid content of cauliflower (depending on the variety) increased by 6- and 15-fold after boiling. Sikora and Bodziarczyk [44] found a 5% decrease in beta-carotene after cooking kale. Similar to vitamin C, biofortification resulted in a protective effect, ensuring a lower loss of total carotenoids in our curly kale study.

For total polyphenols, the steaming process showed the smallest decrease for ‘Redbor F_1_’, while the blanching process was most beneficial for ‘Oldenbor F_1_’, both biofortified with 5,7-diI-8-Q. The boiling process had the most unfavorable effect from all used thermal treatments, resulting in the highest loss of polyphenols for both varieties biofortified with 5,7-diI-8-Q. Compared to the results obtained by Korus [54] and Sikora et al. [46], the loss of polyphenols was much higher. This may be due to the different heat treatment times. Polyphenols can be lost during boiling due to oxidation at high temperatures, or can be leached out into hot water [55]. In addition, other studies showed that different cooking methods had a negative effect on phenolic compounds in vegetables. In the study by Miglio et al. [28], no polyphenols were found after the cooking process of the carrots and broccoli, while the steaming process reduced the content of polyphenols by about 40%. Armesto et al. [56] found that the total phenolic content of curly kale was reduced by all cooking methods (boiling, pressure cooking, microwave steaming).

A number of research studies in the literature have addressed the issue of food safety of iodine biofortification of vegetables. The recommended dietary allowance (RDA) for iodine is 150 µg/day for adults. This varies according to age and sex. During pregnancy and lactation, iodine requirements are higher, up to 220 and 290 µg/day, respectively. In this study, the consumption of curly kale leaves biofortified with 5,7-diI-8-Q provided from 45 to 84 µg and from 90 to 169 µg of iodine from 50 and 100 g portions of the edible product after the heat treatment, respectively. Steaming had the most beneficial effect because it caused the lowest loss of iodine in the plant. Therefore, the amount of iodine in the curly kale was higher than the recommended RDA-I for adults (in 100 g of kale) only after the leaf steaming process had been applied to the biofortified ‘Redbor F_1_’ and ‘Oldenbor F_1_’ varieties. One serving of the heat-treated control curly kale did not meet the daily iodine requirement. We can conclude that, thanks to the biofortification with 5,7-diI-8-Q, although curly kale has been subjected to heat treatment, which causes a decrease in the iodine content of the vegetable, the consumption of 100 g of curly kale provides a higher dose than the RDA.

An important finding of our study was that different dietary regimens significantly affected urinary and fecal iodine concentrations. Based on the WHO recommendations, the nutritional status of iodine in humans is assessed based on its content in the urine [57]. We found the highest content of iodine measured in urine of the group of animals fed a diet containing curly kale ‘Oldenbor F_1_’ biofortified with 5,7-diI-8-Q in the 8th week, as compared to the urine of rodents from the control diet (*AIN-93G*) and the diet containing control curly kale ‘Oldenbor F_1_’ groups. In the 1st week, we found a tendency for a higher level of this microelement in the diet containing curly kale ‘Oldenbor F_1_’ biofortified with 5,7-diI-8-Q in rodents. This confirms that the organic form of iodine is well absorbed from the intestines and improves the nutritional status of iodine in rats. Additionally, it can be assumed that the variety of curly kale can perform an important role in the bioavailability of iodine from the organic form, because we found a tendency for higher levels of iodine in the feces of rats fed the diet containing curly kale ‘Redbor F_1_’ biofortified with 5,7-diI-8-Q. The lack of changes in the iodine content in the 1st week of the experiment between all experimental groups can be explained by the presence in the diet of not only fiber as cellulose but also as a mixture of cellulose and fiber from the curly kale. Also, the presence of polyphenolic compounds could affect the absorption of iodine, as cited below. In the available literature, there are only a few studies concerning the absorption of iodine from vegetables. Piątkowska et al. [58] reported that the concentration of iodine in the urine of rats fed a diet with raw carrot biofortified with the non-organic form of iodine (KI) during agriculture was higher compared to the urine of rats fed a diet with the control carrot. Rakoczy et al. [59] also showed similar results in an animal study in which rodents were fed a diet with lettuce. But they were still lower compared to the control group where the source of iodine was a mineral mix. In our study, we found that the iodine concentration from a diet containing curly kale ‘Oldenbor F_1_’ biofortified with 5,7-diI-8-Q was higher than in the urine of control rats. This again confirms that iodine from kale that during growing was biofortified with an organic form of iodine has a better bioavailability. Our study is the first and innovative in both the chemical composition profile and the bioavailability of iodine to the living organism from iodoquinolines. Tonacchera et al. [60] showed that lettuce, raw carrots, tomatoes, and potatoes were biofortified with iodine salt, with iodine concentrations ranging from 1 to 50 g, via a method involving the administration of an aqueous solution during the growing season—consumption of these vegetables significantly increased iodine concentrations in excreted urine in humans. Similar results were obtained by Baldassano et al. [61], who conducted a study in which humans consumed curly endive biofortified by providing iodine in the form of potassium iodate, applied via foliar spraying during the growing season. The increase in urinary iodine concentration indicated that a higher dose of this element is provided in the diet through biofortified vegetables. Fortification of vegetables with iodine may help to improve the nutritional status of the population with this element.

## 5. Conclusions

The results of this study show that biofortification with 5,7-diI-8-Q resulted in significantly higher levels of iodine in curly kale compared to the vegetable biofortified with KIO_3_ and without biofortification. The thermal processes used in the study (steaming, blanching, boiling) indicate that biofortified curly kale has significantly higher iodine levels compared to thermally treated vegetables without biofortification. The steaming process is the most favorable form of the heat treatment and leads to the lowest iodine loss in kale. Curly kale (‘Oldenbor F_1_’, ‘Redbor F_1_’) enriched with 5,7-diI-8-Q has the lowest iodine loss when compared to that enriched with KIO_3_ and to the control group after heat treatment processes. Additional ingredients of biofortified curly kale were higher than or equal to vegetables without biofortification. Biofortification of curly kale resulted in successful bioavailability and absorption in the digestive tract of the experimental rats. Therefore, the increasing popularity of kale as a superfood in recent years can be considered a potential opportunity to add iodine into the daily diet from this kind of vegetable biofortified with 5,7-diI-8 Q. The results of this research could open the door to new health approaches that focus on expanding access to iodine, especially in regions with limited natural iodine levels. Such an initiative could not only improve overall health but also reduce the risk of thyroid disease, high blood pressure, obesity, type 2 diabetes, and related health problems.

## 6. Patents

The method of biofortification of vegetables in iodine cultivated using a traditional, soilless, and hydroponic method, and the use of 5,7-diiodo-8-quinolinol for biofortification of vegetables with iodine. These are covered under patent application number P.443221 for the compounds (Polish Patent Office; 21 December 2022).

## Figures and Tables

**Table 1:** Ingredients of experimental diets’ compositions.

Ingredient (g·kg^−1^)	C	CO	BO	CR	BR
Corn starch	397.486	524.716	524.896	526.036	525.456
Saccharose	100	100	100	100	100
Casein	200	200	200	200	200
Soybean oil	70	70	70	70	70
Fiber	50	47.02 ^a^	50	50	50
Vitamin mix ^b^	10	10	10	10	10
Mineral mix ^b^	35	35	35 ^c^	35	35 ^c^
Choline	2.5	2.5	2.5	2.5	2.5
TBHQ ^d^	0.014	0.014	0.014	0.014	0.014
Biofortified kale ^e^	-	-	11.2	-	11.1
Control kale ^e^	-	10.75	-	9.98	-

C control diet (*AIN-93G*), CO diet containing control curly kale ‘Oldenbor F_1_’, BO diet containing biofortified curly kale ‘Oldenbor F_1_’, CR diet containing control curly kale ‘Redbor F_1_’, BR diet containing biofortified curly kale ‘Redbor F_1_’. ^a^ 2.92 g of fiber was delivered from control curly kale. ^b^ according to *AIN-93G.* ^c^ mineral mix without iodine; in these diets, the source of iodine was biofortified curly kale. ^d^ *tert*-butylhydroquinone. ^e^ freeze-dried curly kale.

**Table 2:** Daily intake, coverage of recommended dietary allowance for iodine (RDA-I), and hazard quotient (HQ) for intake by adults consuming 50 g and 100 g portions of heat-treated curly kale (f.w.).

Type of Heat Treatment	Cultivar	Daily Intake of I		Coverage of RDA-I		HQ	
		With 50 g of Curly Kale (µg I Day^−1^)	With 100 g of Curly Kale (µg I Day^−1^)	In 50 g of Curly Kale (%)	In 100 g of Curly Kale (%)	For 50 g of Curly Kale	For 100 g of Curly Kale
steaming	Oldenbor F_1__control	4.68 ± 0.08 c	9.35 ± 0.06 c	3.12 ± 0.05 c	6.23 ± 0.10 c	0.000 ± 0.000 a	0.000 ± 0.000 a
	Oldenbor F_1__5,7-diI-8-Q	75.87 ± 0.36 g	151.75 ± 0.47g	50.58 ± 0.000 g	101.16 ± 0.31 g	0.015 ± 0.000 e	0.030 ± 0.000e
	Redbor F_1__control	3.93 ± 0.04 bc	7.85 ± 0.08 bc	2.62 ± 0.03 a	5.24 ± 0.05 bc	0.000 ± 0.000 a	0.000 ± 0.000 a
	Redbor F_1__5,7-diI-8-Q	84.31 ± 0.04 h	168.62 ± 0.73 h	56.21 ± 0.024 h	112.41 ± 0.48 h	0.018 ± 0.000 f	0.037 ± 0.000 f
blanching	Oldenbor F_1__control	2.53 ± 0.03 ab	5.07 ± 0.06 ab	1.69 ± 0.02 ab	3.38 ± 0.04 ab	0.000 ± 0.000 a	0.000 ± 0.000 a
	Oldenbor F_1__5,7-diI-8-Q	74.72 ± 0.71 g	149.46 ± 0.47 g	49.82 ± 0.001 g	99.64 ± 0.95 g	0.015 ± 0.000 e	0.029 ± 0.001 e
	Redbor F_1__control	2.23 ± 0.04 ab	4.53 ± 0.09 ab	1.51 ± 0.03 ab	3.02 ± 0.06 ab	0.000 ± 0.000 a	0.000 ± 0.000 a
	Redbor F_1__5,7-diI-8-Q	65.28 ± 0.07 e	130.56 ± 1.37 e	43.52 ± 0.46 e	87.04 ± 0.91 e	0.011 ± 0.000 c	0.022 ± 0.000 c
boiling	Oldenbor F_1__control	4.13 ± 0.03 bc	8.26 ± 0.06 bc	2.75 ± 0.00 bc	5.50 ± 0.04 bc	0.000 ± 0.000 a	0.000 ± 0.000 a
	Oldenbor F_1__5,7-diI-8-Q	45.10 ± 0.21 d	90.19 ± 0.43 d	30.06 ± 0.02 d	60.13 ± 0.28 d	0.005 ± 0.000 b	0.011 ± 0.000 b
	Redbor F_1__control	0.002 ± 0.000 a	4.10 ± 0.07 a	1.35 ± 0.02 a	2.71 ± 0.05 a	0.000 ± 0.000 a	0.000 ± 0.000 a
	Redbor F_1__5,7-diI-8-Q	72.61 ± 0.71 f	145.22 ± 1.41 f	48.41 ± 0.47 f	96.81 ± 0.940 f	0.014 ± 0.000 d	0.027 ± 0.001 d

Concentrations of daily intake, percentages of RDA-I (recommended daily allowance for iodine), and hazard quotients (HQs) for intake of iodine through consumption of 50 and 100 g portions of two curly kale cultivars (‘Oldenbor F_1_’, ‘Redbor F_1_’) biofortified with 5,7-diiodo-8-quinolinol after thermal treatment (steaming, blanching, boiling). Means followed by the same letters for treatments for each curly kale variety cultivar are not significantly different (*p* > 0.05). Results are shown as mean ± standard deviation (SD).
